# An efficient CRISPR vector toolbox for engineering large deletions in *Arabidopsis thaliana*

**DOI:** 10.1186/s13007-018-0330-7

**Published:** 2018-08-02

**Authors:** Rui Wu, Miriam Lucke, Yun-ting Jang, Wangsheng Zhu, Efthymia Symeonidi, Congmao Wang, Joffrey Fitz, Wanyan Xi, Rebecca Schwab, Detlef Weigel

**Affiliations:** 1 Department of Molecular Biology, Max Planck Institute for Developmental Biology, 72076 Tübingen, Germany; 2 Present Address: Singlera Genomics, Lane 500 Furonghua Road, Pudong, Shanghai, 201318 China

**Keywords:** *Arabidopsis thaliana*, Genome editing, CRISPR/Cas9, Natural variation, NLR genes

## Abstract

**Background:**

Our knowledge of natural genetic variation is increasing at an extremely rapid pace, affording an opportunity to come to a much richer understanding of how effects of specific genes are dependent on the genetic background. To achieve a systematic understanding of such GxG interactions, it is desirable to develop genome editing tools that can be rapidly deployed across many different genetic varieties.

**Results:**

We present an efficient CRISPR/Cas9 toolbox of super module (SM) vectors. These vectors are based on a previously described fluorescence protein marker expressed in seeds allowing identification of transgene-free mutants. We have used this vector series to delete genomic regions ranging from 1.7 to 13 kb in different natural accessions of the wild plant *Arabidopsis thaliana.* Based on results from 53 pairs of sgRNAs targeting individual nucleotide binding site leucine-rich repeat (NLR) genes, we provide a comprehensive overview of obtaining heritable deletions.

**Conclusions:**

The SM series of CRISPR/Cas9 vectors enables the rapid generation of transgene-free, genome edited plants for a diversity of functional studies.

**Electronic supplementary material:**

The online version of this article (10.1186/s13007-018-0330-7) contains supplementary material, which is available to authorized users.

## Background

A central question in biology is how genes direct organismal function and phenotype. Much of the mechanistic knowledge we have today about development and physiology has come from genetic analyses, but these have been usually restricted to a few standard genetic backgrounds. In the model plant *Arabidopsis thaliana*, this has typically been the reference accession Columbia-0 (Col-0) plus a few other popular accessions such as Landsberg *erecta* (L*er*) or Wassilewskija-2 (Ws-2), chosen for convenience, rather than because they are the most typical representatives of their species.

An alternative approach to connecting genotype and phenotype is to exploit intraspecific variation, which has added the benefit of informing about genes and alleles that help organisms to adapt to their environment. This approach has in the past decade been fueled by the rapidly increasing knowledge of genetic variation in hundreds, if not thousands of individuals. In *A. thaliana*, this has culminated in the 1001 Genomes project [1], a major resource for genome wide association analyses (GWAS) [2–5]. However, while GWAS can provide a fast track to the preliminary identification of genetic variants that are likely to underlie phenotypic variation between different individuals, confirmation that the statistically identified variants have a causal role in a specific biological process requires genetic manipulation. Often, the fastest way for obtaining such supporting information is from knockout studies in one of the reference strains [6, 7]. However, that requires the reference strain to have a functional copy of the candidate gene in question. This may not be the case, either because the reference strain has an inactive allele, or because the reference strain lacks the gene all together. It is therefore desirable to carry out equivalent genetic tests in other accessions as well.

The *Streptococcus pyogenes*—derived CRISPR (Clustered Regularly Interspaced Short Palindromic Repeats)/Cas9 (CRISPR-associated protein 9) system has become the method of choice for targeted gene modifications in many organisms, including plants [8–10]. Cas9 can be programmed by a single guide RNA (sgRNA) to induce a DNA double strand break (DSB) at a specific genomic site that is complementary to a 20-base sequence in the sgRNA next to a short Protospacer Adjacent Motif (PAM sequence) [11]. If the DSB is not correctly repaired, short insertions/deletions (indels) or base substitutions can be caused by non-homologous end joining (NHEJ). If the DNA modification is introduced in the germline, the “trigger” transgene can be removed by segregation. That single sgRNAs can induce indels with high frequency is well known [12].

In addition to short indels, CRISPR/Cas9 technology can generate deletions of DNA fragments flanked by target sites for two different sgRNAs. Such larger deletions may be desirable when regulatory sequences need to be removed, or when several tandemly repeated genes need to be inactivated and when truncation of an open reading frame can lead to gain-of-function mutations, as is often the case for nucleotide binding domain leucine-rich repeat (NLR) protein genes [13, 14]. CRISPR/Cas9 induced larger deletions have been widely documented in vertebrates [15–18] and invertebrates [19, 20].

In plants, there have been reports not only of modest sized deletions (under 1 kb) in crops such as rice and tomato [21, 22], and in *Arabidopsis* [23, 24], but also much larger deletions, as large as 120 kb in *Arabidopsis* [24] or 245 kb in rice protoplasts [21]. Unfortunately, such events are rare, and a large number of plants needed to be screened to identify them. Whether they could be inherited is unclear.

We have developed an efficient and easy-to-use super module (SM) destination and sgRNA shuffle-in vector toolbox that facilitates the generation and identification of deletions in plants. We demonstrate its usefulness by targeting 53 different *NLR* genes, aiming for deletions ranging from 1.7 to 13 kb. At least half of the events were inherited independently of the Cas9-sgRNAs. Deletions were obtained in multiple accessions of *A. thaliana*, demonstrating the utility of these vectors for GxG studies.

## Results and discussion

### CRISPR/Cas9 SM-gRNA-shuffle-in vector toolbox

**Fig. 1:**
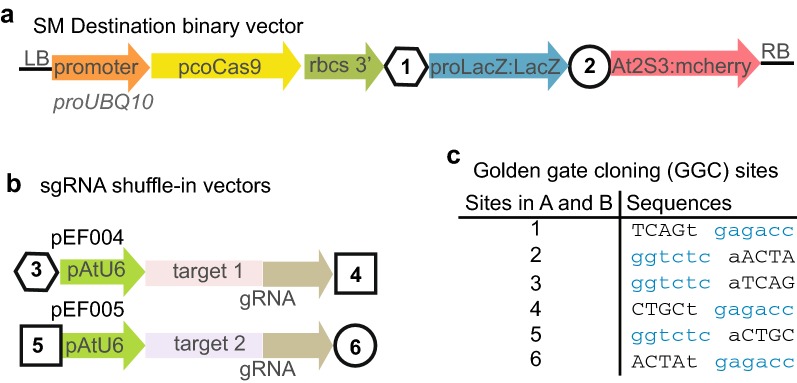
SM (super module) Destination binary vectors and sgRNA shuffle-in vectors. **a** Schematic representation of SM Destination binary vectors. Plant codon optimized *Cas9* (*pcoCas9*) is driven by the promoter of *UBQ10* (*proUBQ10*). Transcriptional termination sequences from rbcS. Blue-white selection strategy with *LacZ* cassette. Seed coat expressed red fluorescence from *At2S3:mcherry* cassette as transgenic plant selection marker. Not drawn to scale. **b** Schematic representation of sgRNA shuffle-in vectors. sgRNA including 20-bp target sequences and shared sgRNA sequences transcribed by *A. thaliana U6* promoter. **c** Overhang sequences used for Golden gate cloning in generating the final binary vectors. The numbers listed in left column are indicated in **a** and **b**

In plants, CRISPR/Cas9 components are typically introduced as transgenes. To confirm that a mutation has been established in the germline, the transgene must be removed by segregation [25]. Our super module (SM) sgRNA-shuffle-in vector (SM CRISPR vector) toolbox facilitates both the generation of transgenic constructs, and the identification of later-generation, transgene-free plants. The toolbox has two parts, the SM destination binary vector (Fig. 1a) and the sgRNA shuffle-in vectors (Fig. 1b).

The SM destination vector was generated by Gibson cloning [26] using the pGreen binary vector pGGZ001 [27], which is Golden gate cloning compatible [28]. The bacterial counter selection *ccdB*/*chloramphenicol*^*R*^ cassette was replaced with the *lacZ* gene driven by the *lacZ* promoter. This enables blue-white selection to monitor replacement of the *lacZ* cassette with sgRNA cassette. A plant codon optimized *Cas9* open reading frame (*pcoCas9*) [29] was combined with a 3′ *rbcS* terminator in the modified pGGZ001 destination vector (Fig. 1a). *Not*I/*BamH*I double restriction sites were engineered in front of the *pcoCas9* ORF sequence, for the insertion of different promoters that can induce *pcoCas9* expression in stage- or tissue-specific manner. For example, we used the *UBQ10* (*AT4G05320*) promoter to drive ubiquitous expression in all organs of *A. thaliana* [30, 31]. To select transgenic plants, we used mCherry expression under the control of the *At2S3* (*AT4G27160*) promoter, which leads to red fluorescence in the seed coat [25]. This allows both positive (T1 generation) and negative selection (T2 generation) of the CRISPR/Cas9 transgene (Additional file 1: Figure S1).

**Fig. 2:**
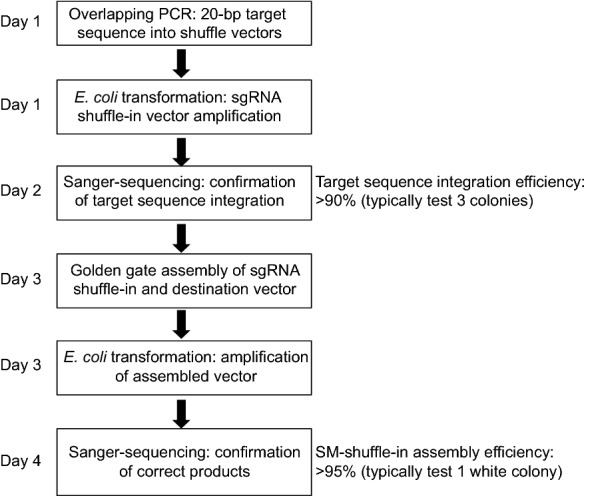
Workflow of using SM vectors

sgRNA shuffle-in vectors were generated based on pGGD000 and pGGE000 vectors [27], which are also Golden Gate cloning compatible. A cassette consisting of the *AtU6* promoter and sgRNA scaffold sequences was cloned into the above vectors. The *proAtU6:target*-*sgRNA* cassette can be tandemly shuffled into the SM destination binary vector by Golden Gate assembly with overhang sequences (Fig. 1c) in a simultaneous digestion/ ligation one tube reaction (Fig. 2).

### Application of the SM-gRNA-shuffle-in toolbox

The 20-bp portion of the sgRNA providing targeting specificity can be engineered into sgRNA shuffle-in vectors by mutagenesis PCR ("Methods" and Fig. 2), and in 90% of cases it was sufficient to test only three colonies to have at least one that was correct. PCR reaction conditions needed to be optimized, such as annealing temperatures and extension time for some primer pairs, depending on the GC content in the 20-bp-target sequence.

Swapping the *proAtU6:target*-*sgRNA* into the SM Destination vectors can be accomplished by Golden gate assembly [28]. White-blue selection is efficient, and over 95% of cases it was sufficient to test only one white colony (after swapping) to get the one harboring correctly assembled plasmids. Because the *pcoCas9* sequence, especially at the 3’ end, was sometimes partially deleted in *E. coli*, it is recommended to confirm the assembled vectors by sequencing. This toolbox is efficient and straightforward to use, and the entire cloning process can be completed in 5 days (Fig. 2). We generated 40 constructs in 1 week.

### Generation of genomic deletions in *Arabidopsis thaliana* natural accessions

Because premature stop codons or frame shifts can sometimes be associated with gain-of-function phenotypes, it may be advisable to partially or completely delete the open reading frame of a gene of interest. We therefore wanted to test whether there are limits to deletion size that can be induced by CRISPR/Cas9.

**Fig. 3:**
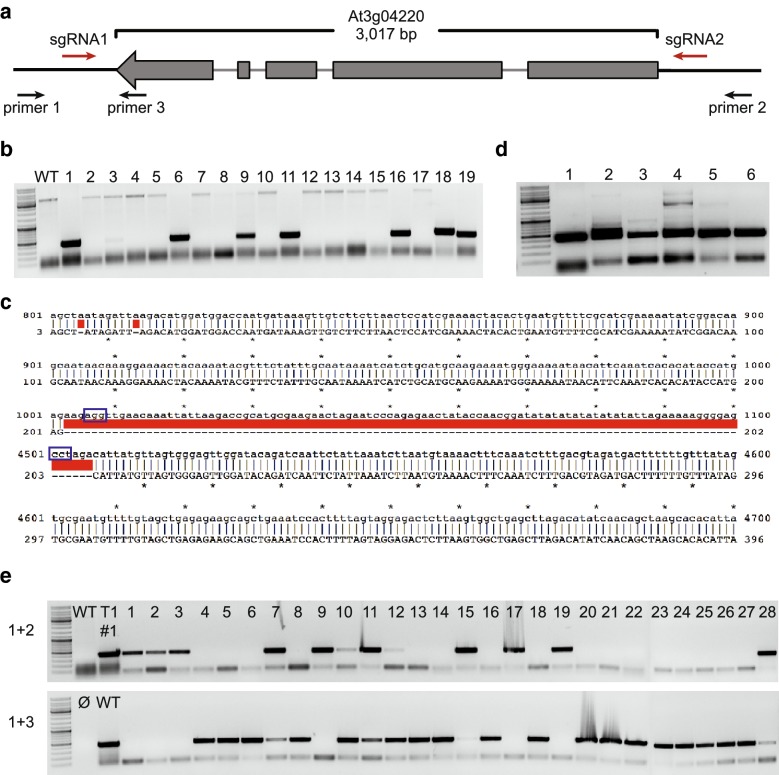
An example of inherited deletions in different accessions. **a** Schematic representation of targeted gene At3g04220 (U17), with locations of sgRNA target sites and primer binding sites for genotyping. **b** Deletions of At3g04220 in Col-0 reference accession, as detected by PCR with primers designated in **a**. Nineteen individual T1 plants were tested. Expected size of PCR product for deletion is 404 bp. **c** Alignment of sequence of PCR product for deletion in **b** (red) with wild-type sequence, which is only partially shown. **d** Deletions of At3g04220 in T1 plants from natural accessions, as detected by PCR; number of pooled plants in square brackets. 1: Col-0 #1 sample in **b**; 2: TueSB30-3 [9]; 3: Nie1-2 [7]; 4: WalHaesB4 [13]; 5: Rue3.1-31 [6]; 6: TueWa1-2, TueV-13, and HKT2.4 [5] (transformation efficiency is low for these 3 accessions). **e** Inheritance of At3g04220 deletion in 28 transgene-free Col-0 T2 plants, progeny of #1 T1 plant in **b**. Several plants show only the mutant band (top), indicating that they are homozygous for the deletion

Fifty-three pairs of 20-bp target sequences were designed with CRISPR-P [32] based on the Col-0 reference genome (http://www.arabidopsis.org/), to target sequences flanking 53 individual *NLR* gene ORFs (Table 1 and Additional file 2: Table S1). Conservation of target sequences was ascertained with polymorphism information from 80 and 1135 natural *A. thaliana* accessions [1, 33] (Additional file 3: Figure S2), to ensure the sgRNAs can function to the same targets in other natural accessions. T-DNAs encoding *proUBQ10:pcoCas9* and sgRNAs were introduced into the Col-0 reference accession (see "Methods") using *Agrobacterium* floral dip method [34]. Seeds carrying the T-DNA constructs were identified directly among the seeds harvested from the *Agrobacterium* treated plants based on red fluorescence from seed coat (Additional file 1: Figure S1a) under a dissecting microscope with dsRed filter (see "Methods"). Three oligonucleotide primers (Additional file 4: Table S2) were designed to genotype these T1 plants for deletions. Primers 1 and 2 were designed to flank the deletion (Fig. 3a, b), and control primers 1 and 3 were designed so as to span one of the two sgRNA target sites, with the binding site of primer 3 inside the expected deletion (Fig. 3a, e). Genotyping for a deletion event was first conducted in pooled T1 plants (pools of 6-25 plants for each target, Additional file 2: Table S1) with different transgene insertions. In the pools in which deletions were detected, individual T1 plants were analyzed.

**Table 1:** Summary of efficiency of inherited deletion events in Col-0 with *UBQ10* promoter driving expression of *pcoCas9*

Targeted genes	53
Genes with deletions in T1 pools	34
Size of deleted fragments	
< 3 kb (1.7–3 kb)	3/6 (50%)
3–7 kb	26/42 (61.9%)
> 7 kb (7–13 kb)	5/5 (100%)
Frequency of deletion events in T1	4.8–78.6%
Genes with inheritance of deletions in T1	17/23
Deletion inheritance in T2 (≤100 T2 plants)	1-90%

With *UBQ10* promoter driving *pcoCas9* (SM Destination vector pRW004, Additional file 5: Table S3), we found deletions in pooled T1 plants for 34 out of the 53 targeted genes. The sizes of deleted fragments ranged from 1.7 to 13 kb (Fig. 3a, Table 1 and Additional file 2: Table S1). The appearance of deletions appeared to be independent of size. Specifically, we found 50% (3/6) of expected deletions under 3 kb, 62% (26/42) of deletions between 3 and 7 kb, and 100% (5/5) of deletions over 7 kb.

Several factors could confound our estimates of successful deletion frequency. First, genotyping PCR might be a factor. The absence of a PCR product diagnostic of a deletion cannot be distinguished from a failed PCR reaction. Second, success and failure rates appear to depend to some extent on the targeted locus as well as the similarity in efficiency of the two sgRNAs. Local genome properties, such as methylation and chromatin compaction, may affect accessibility of sgRNA to target sequences, and DNA-bound transcription factors may block the loading of Cas9 endonuclease. Also, unequal targeting efficiency of the two sgRNAs might cause one of the sites to be consistently mutated before a deletion could occur by simultaneous cutting of both targets by Cas9. Changing one or both of the sgRNAs might be helpful in such cases (Additional file 6: Figure S3). Another potential contributing factor could be incomplete transfer of the *proUBQ10:Cas9* cassette, which is immediately next to the left border (LB) of the T-DNA. The T-DNA LB is less stable than the right border (RB), often causing incomplete integration of adjacent transgene sequences [35]. We tested several loci and found that some individual transgenic plants had incomplete transgene sequences next to the LB, although we did not find a clear correlation between missing LB sequences and lack of target deletions (Additional file 7: Figure S4). To save on later genotyping efforts, it is recommended to ascertain completeness of Cas9 sequences adjacent to LB before testing for target deletions or mutations.

**Fig. 4:**
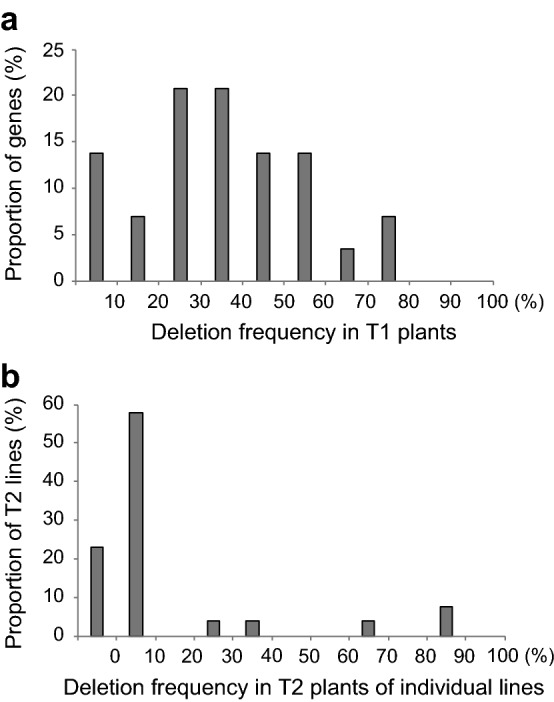
Frequencies of deletions and inheritance in T2 generation. **a** Deletion frequencies in T1 plants for the 34 out of 53 targeted genes where deletions were detected (Additional file 2: Table S1). **b** Frequencies of deletion inheritance in T2 for the 17 genes whose deletions were inherited (Additional file 2: Table S1)

The next question we wanted to answer was how many T1 plants need to be screened for a deletion event. Based on the 34 genes for which we detected deletions, the frequency ranged from 5% (1 out of 21 T1 plants, U52/At4g08450) to 79% (11 out of 14 T1 plants, U7/At1g27170) (Table 1 and Additional file 2: Table S1), with frequency for most genes being between 20 and 40% (Fig. 4a). There was no correlation between the frequency of T1 plants with deletion and the size of deleted fragments.

We also wanted to test how well our toolbox works in other *A. thaliana* accessions. The *UBQ10* promoter drives strong expression in *A. thaliana* accessions (http://jsp.weigelworld.org/AtGenExpress/resources/), and we introduced the construct targeting the At3g04220 gene (U17) into seven natural accessions. In Col-0, we detected in 6 out of 19 T1 plants the expected 3,536 bp deletion (Fig. 3b), which was confirmed by Sanger sequencing (Fig. 3c). The remaining 13 T1 plants were indistinguishable from our untransformed control. Because of lower transformation efficiency in the natural accessions, fewer T1 plants were obtained, but several carried deletions (Fig. 3d), with the frequency of 20 to 40% being similar to the one seen in Col-0 (Additional file 8: Table S4).

### CRISPR/Cas9-induced deletions are stably inherited

As discussed above, DNA fragment deletions have been reported [21–24], but there are few systematic studies of the inheritance of such deletions. We used the same pairs of primers to test for the presence of deletions in transgene-free T2 plants for 23 out of the 34 genes for which deletions had been found in the T1 generation (Table 1 and Additional file 2: Table S1). In a first round of screening, 30 to 50 T2 plants from each individual T1 line with a deletion event were pooled and genotyped by PCR, followed by screening of individual plants if a deletion was detected in the pool. If no inherited deletion could be detected, a second round of screening with 50 to 150 additional T2 plants was performed. In 17 of the 23 tested cases, deletions were recovered in transgene-free T2 plants, with frequencies of 1 to 90%, with most below 10% (Table 1 and Fig. 4b, Additional file 2: Table S1).

Several factors could explain the variation in stable inheritance of deletions. First, in the T1 generation, we tested the somatic tissue of leaves, and while the *UBQ10* promoter drives strong expression of *pcoCas9* in many tissues, it may be less effective in the germline. EC1.2en-EC1.1p [36], an egg-cell specific hybrid promoter, has been reported to increase the frequency of heritable CRISPR/Cas9-induced mutations compared to the more widely expressed *UBQ10* promoter. Second, *UBQ10* promoter activity in the germline may be particularly sensitive to the genomic insertion site of the transgene, explaining large variation in deletion inheritance, such as for At1g63750 (U13 in Additional file 2: Table S1). Some deletions were, however, observed at more consistent frequencies. For example, At3g04220 (U17) deletions were often inherited (35 to 83% in progenies from 6 T1 lines, Additional file 2: Table S1), while At5g45250 (U36) deletions were only rarely inherited (2% in 3 T1 lines Additional file 2: Table S1). In the U17 and U30 lines, we found homozygous and heterozygous mutants as well as wild-type progeny. In the other lines, most progeny was wild type, and only one or two plants were either homozygous or heterozygous for the deletions. For example, two out of 34 T2 plants for At4g33300 (U24, Additional file 2: Table S1) were both homozygous for the deletion, and the rest was wild type (Additional file 2: Table S1). Based on these analyses, we conclude that on the order of 30 T1 plants are in many cases sufficient to obtain heritable deletions, but a higher number of T2 transgene-free plants, at least 100, is advisable to obtain heterozygous or homozygous mutants.

## Conclusions

In summary, we have set up an efficient toolbox that facilitates knocking out genes or deleting DNA fragments in *A. thaliana*. Based on 53 targeted *NLR* genes, we detected deletions of up to 13 kb in somatic tissue of transgene-carrying T1 plants in more than half of the cases. Although we cannot exclude that deletions may appear in the T2 generation even if none were detected in T1 plants, somatic deletions in T1 plants with *UBQ10* promoter driving *pcoCas9* are a reasonable predictor of heritable deletions in transgene-free T2 plants. With our tested constructs, about three quarter (17/23) of constructs with T1 deletions produced also T2 plants with deletions. Finally, we have shown that the success rate in natural *A. thaliana* accessions is similar to that in the Col-0 reference accession.

## Methods

### Plasmid propagation

Plasmids with *pcoCas9* were propagated in *E. coli* strain DH5α at 30 °C. Bacteria containing binary vectors and sgRNA shuffle-in vectors were grown on LB plates with spectinomycin (50 µg/mL) plus X-gal (50 µg/mL) or ampicillin (50 µg/mL), respectively.

### Construction of Cas9 destination binary vectors and sgRNA shuffle vectors

The pGGZ001 plasmid [27] was used as starting point for constructing SM Destination binary vectors. In pGGZ001, there are two *Bsa*I sites flanking the *ccdB/chloramphenicol*^*R*^ genes. The four-nucleotide overhangs next to each *Bsa*I site were introduced by two rounds of overlap PCRs (Fig. 1c). A *LacZ* promoter*:LacZ* fragment was PCR amplified from pSE7 [37] and cloned between the *Hind*III and *Bcu*I sites of pGGZ001 to replace the *ccdB/Chloramphenicol*^*R*^ cassette. The *At2S3:mCherry*-*terminator* cassette was amplified from pRW003 [25] and inserted into an *Xba*I site next to the *LacZ*α cassette. We modified a *Cas9* (*pcoCas9*) construct with potato IV2 intron and nuclear localization signal that had been codon optimized for both monocotyledonous and dicotyledonous plants [29] (HBT-pcoCas9, Addgene plasmid #52254) by introducing synonymous mutations that altered 7 out of 9 *Bsa*I recognition sites. A *Not*I/*BamH*I restriction site was added at the 5′ end of *pcoCas9*. *pcoCas9* sequences were fused with *rbcS* 3′ transcriptional terminator sequences using Golden Gate cloning [28]. The *pcoCas9*-*rbcS* terminator cassette was added by Gibson assembly (NEB, Ipswich, USA) [26]. *proUBQ10* promoter was inserted in front of *pcoCas9* with *Not*I/*BamH*I.

Starting material for sgRNA shuffle-in vectors were pGGD000 and pGGE000 [27]. A transcription unit containing the *AtU6* promoter (with “G” at the end to enhance transcription), a 19-bp sgRNA sequence and a scaffold gRNA sequence [29] was generated using gene synthesis (GeneArt Gene Synthesis, Thermo Fisher Scientific Life Technologies, Regensburg, Germany). Two *Bsa*I sites flank the cassette and overlapping PCR was used to place different four-nucleotide overhangs (Fig. 1c) next to the *Bsa*I sites.

### sgRNA sequence design and integration of sgRNA into shuffle vectors

Protospacer target sequences were designed with CRISPR-P [32]. In most cases, the suggestions with the highest scores were used. In cases where there was only a small number of automatic suggestions, sequences were chosen manually. Target sequences were inserted into the sgRNA shuffle-in vectors using overlapping PCR with primers that contained 20-bp vector sequences plus 20-bp guide RNA sequences. For example, forward primer with 5′ N_V1_NNNN_V17_N_V18_N_V19_N_V20_ + 20 bp-sgRNA, and reverse primer with 20 bp-sgRNA + N_V21_N_V22_N_V23_N_V24_NNNN_V40_ 5′ were used for inserting a 20 bp-sgRNA between positions N_V20_ and N_V21_ of vector sequence N_V1_NNNNNNN_V20_N_V21_NNNNNN_V40_. The overlapping PCR reaction (10 μl) was set up as follows (final concentrations in brackets): Q5 Hi-Fidelity polymerase reaction buffer (1x, Thermo Scientific), dNTPs (200 μM), forward and reverse primers (0.2 μM each), ~60 ng vector as template, 0.1 μl Q5 polymerase, and ddH_2_O to 10 μl. PCR was according to the manual for Q5 polymerase (Thermo Scientific). We added 0.8 μl 10 x FD buffer and 0.2 μl *Dpn*I (Thermo Scientific) to the final product, and the mix was directly transformed into *E. coli* strain DH5α. Three single colonies were cultured, and plasmids extracted using the GeneJET Plasmid Miniprep Kit (Thermo Scientific), followed by Sanger-sequencing to confirm sgRNA integration. sgRNA sequences and vectors are listed in Additional file 5: Table S3.

### Assembly of SM-sgRNA shuffle-in vector

The sgRNA expression units were assembled into SM Destination vectors using Golden gate cloning [28]. Restriction/ligation reactions (15 μl) were set up as follows: 1× FastDigest buffer (Thermo Scientific, Waltham, MA, the USA), 1.0 mM ATP, 10 U of FD-*Eco31*I (*Bsa*I, Thermo Scientific), 35 U of T4 DNA ligase (Thermo Scientific), 100 ng of SM Destination vector plasmid, 25 ng of each sgRNA shuffle-in vector. Reactions were incubated for 50 cycles (37 °C, 3 min; 16 °C, 5 min), followed by 50 °C for 5 min and 80 °C for 10 min. The reaction product was directly used for *E. coli* transformation, with antibiotics of Spectinomycin (Sigma) and X-Gal (X-galactopyranoside, Sigma). White colonies were grown in liquid culture (usually one) overnight and plasmids were extracted using the GeneJET Plasmid Miniprep Kit (Thermo Scientific). We confirmed integration of sgRNA units and the completeness of other components such as *pcoCas9* sequences with Sanger sequencing.

### Plant material and growth conditions

Non-reference *A. thaliana* accessions TueSB30-3, Nie1-2, WalHaesB4, Rue3.1-31, TueWa1-2, TueV-13, and HKT2.4 have been described [38]. Seeds were surface sterilized with 75% ethanol plus 0.005% TritonX-100, and germinated on soil. Plants were germinated and cultivated in growth rooms at a constant temperature of 23 °C (temperature variability about ± 0.1 °C), air humidity at 65% and long-day conditions (16 h day length), with light (125 to 175 μmol m^−2^ s^−1^) provided by a 1:1 mixture of Cool White and Gro-Lux Wide Spectrum fluorescent lights (Luxline plus F36 W/840, Sylvania, Germany).

### Transgenic plants

The floral dip method [34] was used to transform plants, with *Agrobacterium tumefaciens* strain ASE [39] at an OD_600_ of 0.7 to 1. Presence and absence of transgenes in seeds was ascertained under a Leica MZFLIII Fluorescence Stereomicroscope (Leica, Wetzlar, Germany) with the setting of dsRed filter for the filter system FLUOIII. Luminescence light was provided by Light Engine Sola 365 SM II und Zubehör (Lumencor, Beaverton, OR, the USA).

### Genotyping

Plant genomic DNA was extracted with a modified CTAB method [40]. *Taq* polymerase (NEB) or Q5 High-Fidelity DNA polymerase (Thermo Scientific) were used for PCR amplification to detect deletion events. Annealing temperatures were tested and optimized for each pair of genotyping primers, with extension time kept short enough to favor amplification of fragments carrying deletions under 1 kb, but not the original, non-deleted fragment. T7E1 (T7 Endonuclease I, NEB) was used for identification of mutations at individual sgRNA target sites [41]. DNA fragments were amplified from genomic DNA using a pair of primers spanning the targets with Q5 High-Fidelity DNA Polymerase (Thermo Scientific). The PCR product was purified using PCR Purification Column (Qiagen, Hilden, Germany) and concentration was determined with a Nanodrop 2000 Spectrophotometer (Thermo Scientific). 100 ng of purified PCR product was denatured-annealed under the following conditions: 95 °C for 2 min, ramp down to 85 °C, at 2 °C/s, followed by ramping down to 25 °C at 0.1 °C/s. Annealed PCR products were digested with 5 U of T7E1 for 20 min at 37 °C. The T7E1-digested products were separated on a 2% agarose gel. A similar amount of substrate DNA without T7E1 incubation was used as a negative control.

## Additional files

**Additional file 1: Figure S1.** Fluorescence-based strategy for selection of transgenic plants. **a.** Fluorescent, transgenic T1 seed for At3g04220 deletion (white arrow). **b.** Non-fluorescent, transgene-free T2 seeds for At3g04220 gene deletion, line 5 (white arrows indicate a cluster of non-fluorescent seeds).

**Additional file 2: Table S1.** Frequency of deletion and inheritance for 53 targeted genes in Col-0 with *UBQ10* promoter driving *pcoCas9*.

**Additional file 3: Figure S2.** Conservation of dual sgRNA target sequences and genotyping primers for At3g04220 gene deletion among natural accessions. “ref”, *A. thaliana* reference genome sequence. Sequence alignments were extracted from the genome matrix of 80 accessions [33, http://1001genomes.org/data/MPI/MPICao2010/releases/current/genome_matrix/TAIR10_genome_matrix_2012_03_13.txt.gz] using AWK, rotated by 90° and converted to HTML using Perl. Variants and uncalled sites were highlighted using CSS. **a.** Sequence alignment for the two target sites (Chr3:1108991..1109010 and Chr3:1115509..1115528) among 80 accessions [33]. **b.** Sequence alignment of locations of oligos for genotyping (Chr3:1108776..1108801 and Chr3:1112691..1112705) among accessions. “ref”, *A. thaliana* reference genome sequence.

**Additional file 4: Table S2.** sgRNA target sequences and oligonucleotides for genotyping.

**Additional file 5: Table S3.** Overview of vectors.

**Additional file 6: Figure S3.** Example showing simultaneous sgRNA efficiency in generating deletion in *ACD6* gene. **a.** Schematic representation of *ACD6* (At4g14400) gene structure and locations of sgRNA target sites and binding sites for genotyping primers (indicated by black numbers on top and the position of primer below). **b.** PCR and T7E1 assays showing mutations only at the target site of sgRNA1 but not sgRNA2. The left-most lane next to DNA ladder (Mix) is a wild-type control. **c.** PCR assay revealing deletion events with the combination of sgRNA1 and sgRNA3. The left-most lane next to DNA ladder (1 kb) is a wild-type control.

**Additional file 7: Figure S4.** PCR analyses of the T-DNA left border to determine completeness of integration of the *proUBQ10:Cas9* expression cassette. **a.** Schematic representation of vectors integrated in the genome. Three fragments were PCR amplified. **b.** PCR products. The first lane (p) is the positive control with the SM destination vector as template. mCherry positive plants for four different targets were tested. Red stars indicate plants with target deletions. DNA ladder MIX was used for P1, and 1 kb ladder for P2 and P3 regions. A 404-bp genomic region from the actin gene AT2G37620 was used as the internal control for DNA quality (fourth panel labeled with CK). Samples for different gene loci are separated by lanes with DNA size markers (M). Arrows point to 1 kb for the first 3 panels and 500 bp for the fourth panel (CK).

**Additional file 8: Table S4.** Frequency of At3g04220 gene deletions in natural accessions.
