# Supplementary figures and images for: An efficient CRISPR vector toolbox for engineering large deletions in Arabidopsis thaliana
Source: Plant Methods. 2018 Aug 2;14:65. doi: 10.1186/s13007-018-0330-7 (PMC6071326; doi:10.1186/s13007-018-0330-7)

**a**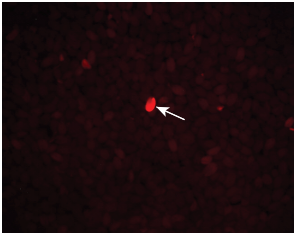**b**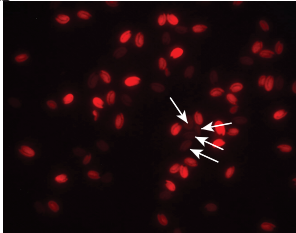

Supplement: Supplementary file 1 — Additional file 1: Figure S1. Fluorescence-based strategy for selection of transgenic plants. a. Fluorescent, transgenic T1 seed for At3g04220 deletion (white arrow). b. Non-fluorescent, transgene-free T2 seeds for At3g04220 gene deletion, line 5 (white arrows indicate a cluster of non-fluorescent seeds). [file 13007_2018_330_MOESM1_ESM.pdf]

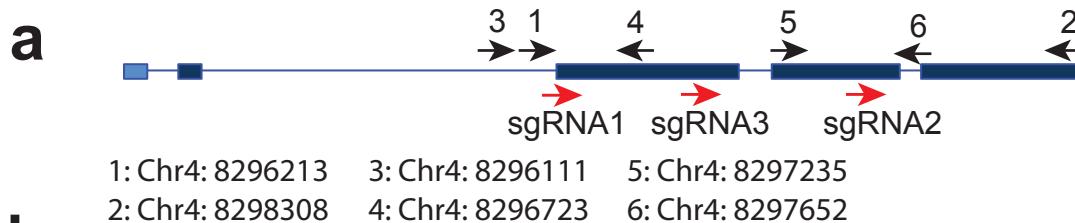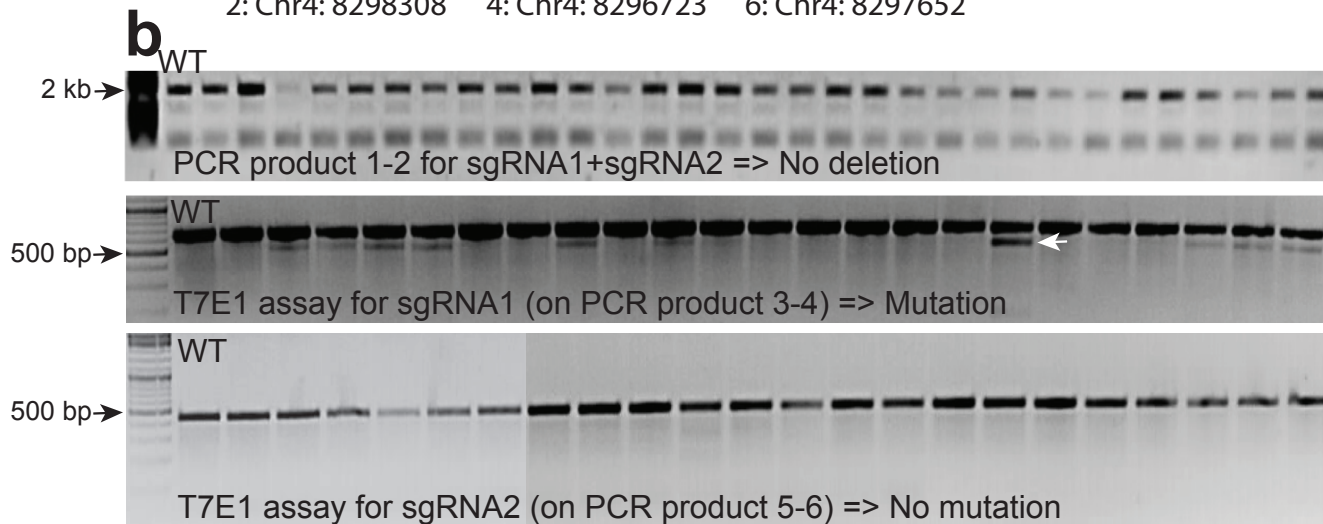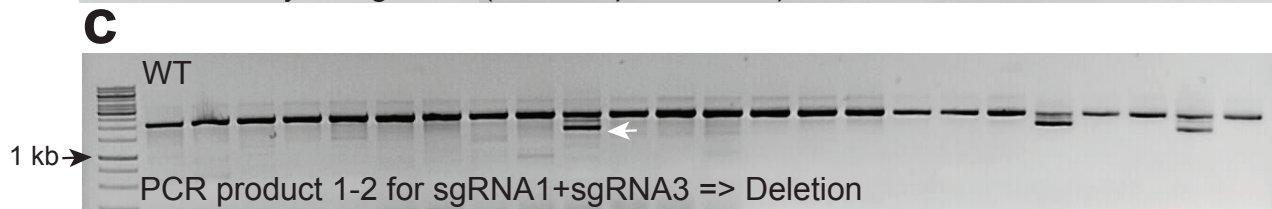

Supplement: Supplementary file 6 — Additional file 6: Figure S3. Example showing simultaneous sgRNA efficiency in generating deletion in ACD6 gene. a. Schematic representation of ACD6 (At4g14400) gene structure and locations of sgRNA target sites and binding sites for genotyping primers (indicated by black numbers on top and the position of primer below). b. PCR and T7E1 assays showing mutations only at the target site of sgRNA1 but not sgRNA2. The left-most lane next to DNA ladder (Mix) is a wild-type control. c. PCR assay revealing deletion events with the combination of sgRNA1 and sgRNA3. The left-most lane next to DNA ladder (1 kb) is a wild-type control. [file 13007_2018_330_MOESM6_ESM.pdf]

**a**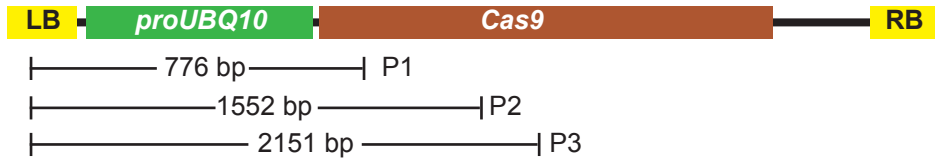**b**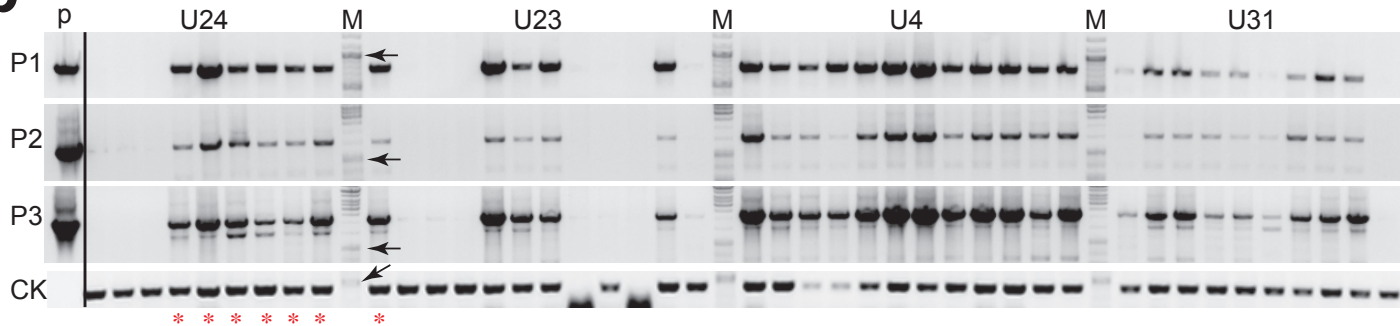

Supplement: Supplementary file 7 — Additional file 7: Figure S4. PCR analyses of the T-DNA left border to determine completeness of integration of the proUBQ10:Cas9 expression cassette. a. Schematic representation of vectors integrated in the genome. Three fragments were PCR amplified. b. PCR products. The first lane (p) is the positive control with the SM destination vector as template. mCherry positive plants for four different targets were tested. Red stars indicate plants with target deletions. DNA ladder MIX was used for P1, and 1 kb ladder for P2 and P3 regions. A 404-bp genomic region from the actin gene AT2G37620 was used as the internal control for DNA quality (fourth panel labeled with CK). Samples for different gene loci are separated by lanes with DNA size markers (M). Arrows point to 1 kb for the first 3 panels and 500 bp for the fourth panel (CK). [file 13007_2018_330_MOESM7_ESM.pdf]
