# Supplementary material for: An efficient CRISPR vector toolbox for engineering large deletions in Arabidopsis thaliana
Source: Plant Methods. 2018 Aug 2;14:65. doi: 10.1186/s13007-018-0330-7 (PMC6071326; doi:10.1186/s13007-018-0330-7)
Supplement: Supplementary file 3 — Additional file 3: Figure S2. Conservation of dual sgRNA target sequences and genotyping primers for At3g04220 gene deletion among natural accessions. “ref”, A. thaliana reference genome sequence. Sequence alignments were extracted from the genome matrix of 80 accessions [33, http://1001genomes.org/data/MPI/MPICao2010/releases/current/genome_matrix/TAIR10_genome_matrix_2012_03_13.txt.gz] using AWK, rotated by 90° and converted to HTML using Perl. Variants and uncalled sites were highlighted using CSS. a. Sequence alignment for the two target sites (Chr3:1108991..1109010 and Chr3:1115509..1115528) among 80 accessions [33]. b. Sequence alignment of locations of oligos for genotyping (Chr3:1108776..1108801 and Chr3:1112691..1112705) among accessions. “ref”, A. thaliana reference genome sequence. [file 13007_2018_330_MOESM3_ESM.pdf]

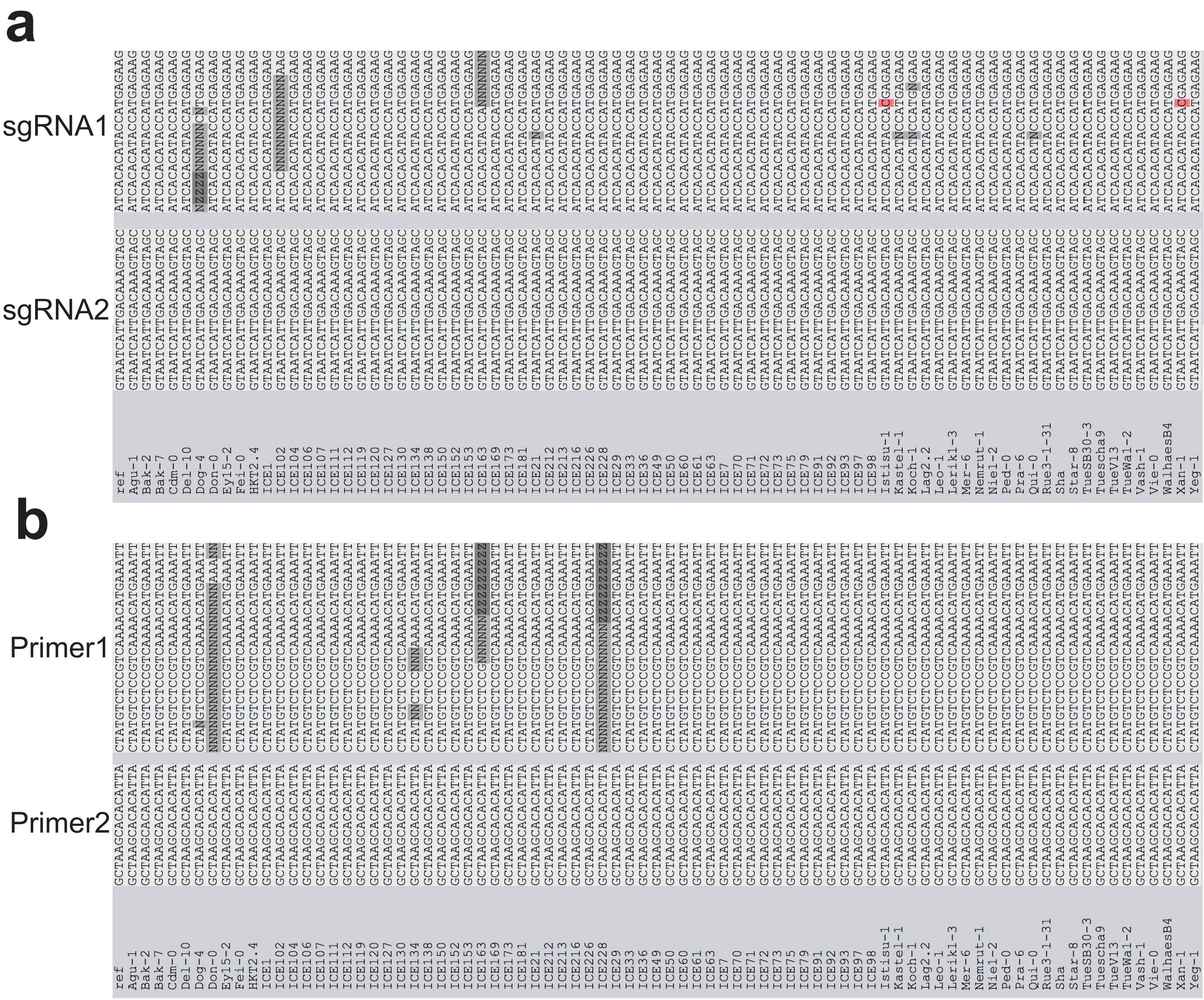
a
sgRNA1
ATCACACATACCATGAGAAG
sgRNA2
GTAATCATTGACAAAGTAGC
b
Primer1
CTATGTCTCCGTCAAAACATGAAATT
Primer2
GCTAAGCACACATTA
ref
Agu-1
Bak-2
Bak-7
Cdm-0
Del-10
Dog-4
Don-0
Ey15-2
Fei-0
HKT2.4
ICE1
ICE102
ICE104
ICE106
ICE107
ICE111
ICE112
ICE119
ICE120
ICE127
ICE130
ICE134
ICE138
ICE150
ICE152
ICE153
ICE163
ICE169
ICE173
ICE181
ICE21
ICE212
ICE213
ICE216
ICE226
ICE228
ICE29
ICE33
ICE36
ICE49
ICE50
ICE60
ICE61
ICE63
ICE7
ICE70
ICE71
ICE72
ICE73
ICE75
ICE79
ICE91
ICE92
ICE93
ICE97
ICE98
Istisu-1
Kastel-1
Koch-1
Lag2.2
Leo-1
Lerik1-3
Mer-6
Nemrut-1
Niel-2
Ped-0
Pra-6
Qui-0
Rue3-1-31
Sha
Star-8
TueSB30-3
Tuescha9
TueV13
TueWal-2
Vash-1
Vie-0
WalhaesB4
Xan-1
Yeg-1
